# Characterization and Performance Enhancement of Cement-Based Thermoelectric Materials

**DOI:** 10.3390/polym14122311

**Published:** 2022-06-07

**Authors:** Ruchita Jani, Niall Holmes, Roger West, Kevin Gaughan, Xiaoli Liu, Ming Qu, Esther Orisakwe, Lorenzo Stella, Jorge Kohanoff, Hongxi Yin, Bartlomiej Wojciechowski

**Affiliations:** 1 School of Civil and Structural Engineering, Technological University Dublin, D07 EWV4 Dublin, Ireland; d18126665@mytudublin.ie (R.J.); bartlomiej.wojciechowski@tudublin.ie (B.W.); 2 Department of Civil, Structural and Environmental Engineering, Trinity College Dublin, D02 PN40 Dublin, Ireland; rwest@tcd.ie; 3 School of Multidisciplinary Technologies, Technological University Dublin, D07 EWV4 Dublin, Ireland; kevin.gaughan@tudublin.ie; 4 Lyles School of Civil Engineering, Purdue University, West Lafayette, IN 47907, USA; liu2251@purdue.edu (X.L.); mqu@purdue.edu (M.Q.); 5 Atomistic Simulation Centre, School of Mathematics and Physics, Queen’s University Belfast, University road, Belfast BT7 1NN, UK; e.orisakwe@qub.ac.uk (E.O.); l.stella@qub.ac.uk (L.S.); 6 School of Chemistry and Chemical Engineering, Queen’s University Belfast, Stranmillis road, Belfast BT9 5AG, UK; 7 Instituto de Fusion Nuclear “Guillermo Velarde”, Universidad Politecnica de Madrid, 28040 Madrid, Spain; j.kohanoff@upm.es; 8 International Centre for Energy, Environment & Sustainability, Washington University in St Louis, St Louis, MO 63130, USA; hongxi.yin@wustl.edu

**Keywords:** cement composites, thermoelectrics, Seebeck coefficient, electrical conductivity, thermal conductivity

## Abstract

Thermoelectric materials enable the direct conversion of thermal to electrical energy. One application of this is ambient heat energy harvesting where relatively stable temperature gradients existing between the inside and outside of a building could be utilized to produce electricity. Buildings can thus change from energy consumers to energy generators. This could ultimately help reduce the surface temperatures and energy consumption of buildings, especially in urban areas. In this paper, research work carried out on developing and characterizing a cement-based thermoelectric material is presented. Cement-based samples are doped with different metal oxides (Bi_2_O_3_ and Fe_2_O_3_) to enhance their thermoelectric properties, which are defined through their Seebeck coefficient, electrical conductivity and thermal conductivity. The study also discusses the positive impact of moisture content on the electrical conductivity

## 1. Introduction

Urbanization is increasing rapidly worldwide and so is the impact of anthropogenic activities. According to an estimate by the United Nations urbanization projections as of 2018, 55% of the world’s population now resides in urban areas [1]. It has resulted in the urban built environment replacing a considerable part of the natural landscape. Pavements, roads and buildings absorb incident solar radiation leading to a 10–20 °C rise in their surface temperature as compared to their surroundings in summer [2]. This phenomenon where the surface temperature of the built environment leads (by re-emitting absorbed radiation at night) to the surrounding air becoming warmer than its nearby rural areas is termed as the Urban Heat Island (UHI) effect [3]. Variation in building heights and sizes in urban areas generates shadows and uneven heat distribution adding to the thermal difference observed during the UHI phenomenon [4]. With pavements and buildings comprising 30–39% and 20% of urban areas, respectively, 60% of urban surface area is now covered by low-albedo and heat-absorbing materials [5,6,7]. The overall energy demands are likely to increase in cities, for adapting the additional heating and cooling loads as a result of climate change and the UHI effect. It has been reported in the literature [8] that cooling demands during summer months are higher in urban areas. However, while efforts to mitigate the UHI effect were successful in reducing the surface temperature of building, its overall effect was limited [9]. Other heat-harvesting technologies such as photovoltaics, thermoelectrics, periodic kinetic, EM wave and airflow require changes in ground level design to integrate them into existing buildings and pavements. The limited power available and the complexities involved in their operation have restricted further application [10,11].

The outdoor air and the indoor air form a relatively stable temperature gradient [12] so thermal energies from these gradients could be captured and converted into electricity using the Thermoelectric (TE) phenomenon. The heat could be utilized to generate movements of electron–hole carriers by using cement-based thermoelectric materials. They can be a useful route to harness absorbed thermal energy in buildings as they could be easily integrated into existing building envelopes through external surfaces or used as a construction material in new buildings [13]. The implementation of cement-based TE materials could lead to a more bearable urban climate and mitigate the UHI effect. It could harvest the waste heat stored in buildings and pavements by converting it into a useful form of energy. Buildings can be converted from being energy consumers to energy harvesters, thus making them more sustainable.

The figure of merit (ZT, Equation (1)) is a dimensionless parameter used to demonstrate the performance of a Thermoelectric (TE) material, where *S*, *σ*, *κ* and *T* represent the Seebeck coefficient, Electrical Conductivity (EC), Thermal Conductivity (TC) and absolute temperature, respectively. For practical applications, a TE material would require its ZT to be greater than 1 [14]. To maximize performance, TE materials require a high electrical conductivity and Seebeck coefficient and a low thermal conductivity to minimize or reduce thermal shortening [15].

(1) $$\mathrm{ZT}=\left(\frac{S^{2}\;\times \;\sigma }{\kappa }\right)T.$$

TE percolation behavior in cement-based materials was observed by Sun and co-workers [16,17] and has grown in interest since. A widely used commercial TE material, bismuth telluride, was mixed in a CFRC composite (as a powder and a coating), and electric polarization was observed as a result, leading to a Seebeck coefficient of 35.5 µV/°C [18]. Wei et al. added micro-sized Fe_2_O_3_ and Bi_2_O_3_ metal oxides to cement and found the Seebeck coefficient to be 92.57 and 100.28 µV/°C, respectively, for a 5 wt.% concentration of the metal powders in the cement matrix [19]. However, the electrical and thermal conductivity of the resulting samples were not studied [19]. The use of nano-sized ZnO and Fe_2_O_3_ powders in combination with cement and silica fume led to high Seebeck coefficient values of 3300 and 2500 µV/°C, respectively [20]. Ca_3_Co_4_O_9_ obtained by synthesis of calcium carbonate and cobalt oxide combined with CFRC-based cement composite generated a Seebeck coefficient of 58.6 µV/°C at 3 wt.% by mass in cement [21]. Pyrolyzed carbon fibers were combined with micro-sized Fe_2_O_3_ particles and cement by Wei et al. This combination led to a high power factor of around 2.08 µW/m^−1^K^−2^ and the highest reported ZT value of 3.11 × 10^−3^ for a cement-based TE material [13]. MnO_2_ powder was synthesized in a laboratory by Tao et al. which resulted into a nanorod-like structure. It was then combined in the cement matrix along with silica fume, and this combination led to a high Seebeck coefficient of 3085 µV/°C, but electrical and thermal conductivities were comparatively low [22]. Ghahari et al. introduced ZnO and aluminum-doped ZnO nanoparticles into the cement matrix. The resulting composite consisting of nano-ZnO helped increase EC values and limit TC values but there was an insignificant increase in the Seebeck coefficient [23]. While additives such as metal oxide powders, carbon and steel fibers, graphite and nanomaterials have improved the thermoelectric performance of cements, it is often difficult to determine the ideal dosage to optimize EC values and Seebeck coefficients [24]. This is further complicated by the relationship and dependency between thermal and electrical conductivity where an increase in the latter will often result in an increase in the former, which is an undesired effect. In addition, at high temperatures and with even small amounts of inhomogeneity, inaccuracies in the characterization of TE materials can occur [25] which makes repeatability of results difficult to achieve in practice [26].

Whilst carrying out a detailed literature review of the various cement-based thermoelectric materials that have been developed to date, certain crucial findings were observed that helped identify the most suitable methods for measuring thermoelectric properties of such materials. The comparison of the different methodologies and details of the electrical contacts and the sample dimensions used for each study is presented in Table 1. It is important to note that except for the study carried out by Ghahari, Ghafari and Lu [23], all the other studies measured the electrical conductivity of the samples using a DC method. It has been reported in the literature that fast-switching DC or AC is the ideal method for measuring the electrical conductivity of cementitious materials [27,28], because cementitious materials are inclined to store electrical charge and thus generate an undesirable polarization effect if electrical resistance is measured using DC signals [29]. Apart from that, a combined effect of a resistive voltage component and a Seebeck voltage component is observed due to subjecting the sample to elevated temperatures. The result is the measurement of two different voltages which can only be differentiated and eliminated if the AC signal is used for electrical resistance measurement at elevated temperatures.

When conventional TE materials are subjected to high temperatures for characterization purposes, inaccuracies in the properties measured are reported to be as high as 50%. These become a bigger concern when samples of different sizes are used for TE characterization and measured on an individual basis [25,26]. Ideally, all samples are supposed to be subjected to similar conditions (especially in terms of sample size and temperature) for their TE characterization to reduce errors in the measurement, however, the majority of the studies described in the literature have used samples of different sizes for different measurements and the measurement conditions were also found to be inconsistent. The Seebeck coefficient is not generally measured for cementitious materials and therefore, whilst doing the TE characterization, methods applicable to semiconductors are considered. This makes it difficult to gauge whether the obtained values are reliable or not without carrying out a comprehensive analysis on applying them to dynamic cementitious materials.

A lot of research has been carried out to determine the magnitude of the TE phenomenon observed at varying proportions of additives and different operating temperatures for cement-based TE materials. However, the following remains outstanding:

▪ What is the duration of the TE phenomenon observed in enhanced cement-based TE materials?
▪ Was the TE observed in dry or saturated samples?
▪ Does the level of sample hydration affect the TE phenomenon?

To date, just one study by Wei et al. has investigated the impact of moisture on the Seebeck coefficient and electrical conductivity of enhanced cement composites [36]. They found that the observed TE phenomenon can be attributed to a high moisture content in the sample, which decreased when it was dried. The materials tested to date for enhancing TE behavior in cement have been tried only in a laboratory environment and are yet to be tested in the dynamic real environment. Thus, there is more in-depth analysis required in studying the TE performance of enhanced TE cement materials to see whether they can be applied to produce thermoelectric power in building envelopes or not.

Here, the thermoelectric properties of cement-based materials doped with micro-Fe_2_O_3_ and Bi_2_O_3_ additives are presented following a study to improve the stability of results obtained.

## 2. Materials and Methods

### 2.1. Materials

Cement samples were prepared using a 42,5 R CEM I rapid hardening cement. It had finer particle size compared to normal CEM II cement. The bismuth trioxide powder used had a purity of 99.5% and a maximum particle size of 50 microns. The ferrous oxide powder used had 95% of its particles of size less than 53 microns. The concentration of metal oxide powders used in the cement mix was 5% of weight by mass of cement for both samples. No additional aggregates were used in preparing the mixture. A water to cement (w/c) ratio of 0.45 was used for all samples. The chemical composition of the batch of CEM I cement used for sample preparation was obtained from its manufacturer and is presented in Table 2.

### 2.2. Sample Preparation and Curing

Three sets of samples were prepared, one was the control sample which consisted of only cement and water mixed with 0.45 as w/c ratio. The other two sets of samples were made of 5% Bi_2_O_3_ and 5% Fe_2_O_3_ weight by mass of cement, respectively. The dry contents were blended appropriately in a container and thereafter the required amount of water was added to form a wet mix using an automatic mortar mixer. The prepared mixture was poured into a stainless-steel mold of size 160 × 40 × 40 mm^3^ and placed on a vibrating table to remove air bubbles from the wet mixture. The samples for thermal conductivity tests had a diameter of 100 mm and height of 200 mm. The prepared mix was allowed to set and solidify in the mold for 24 h and was then demolded. Samples were later exposed to water in a curing tank for a period of 7 days. The curing tank temperature was maintained at 20 ± 1°.

### 2.3. Characterization Techniques

#### 2.3.1. Seebeck Coefficient Test

**Figure 1:**
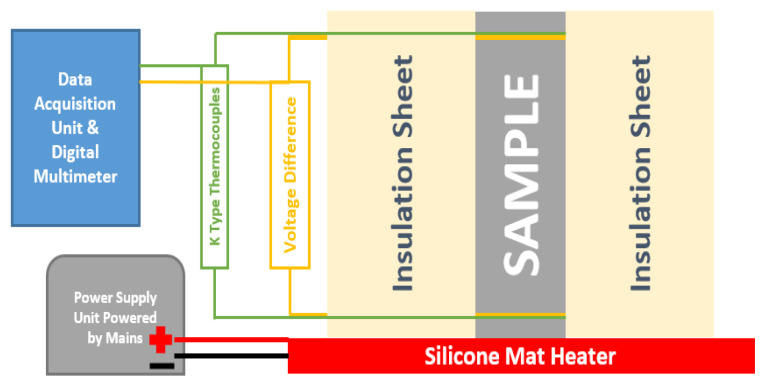
Experimental setup for Seebeck coefficient measurement.

The experimental setup used for measuring Seebeck coefficient was assembled in the lab such that it can measure the voltage difference generated as a result of subjecting the prepared cement sample to a fixed temperature gradient. It consisted of a silicone mat heater connected to a DC power supply unit powered by the mains. One of the square ends (40 mm × 40 mm) of the sample was heated by the silicone mat heater while it was enclosed on all sides using an insulation material having a thermal conductivity of 0.022 W/m-K. K type thermocouples were embedded into the samples during the casting process to know the temperature distribution across the sample length while subjecting it to a temperature gradient. The opposite square of the heated side (40 mm × 40 mm) was subjected to ambient temperature. Temperature sensors (K type) were also attached to the sample at the two ends which were directly subjected to the heating plate and the ambient temperature. The experimental setup used for Seebeck tests is shown in Figure 1. When the arrangement was put in place on the heater, weights were applied to ensure adequate thermal contact existed between the sample and the heater. The sample surrounded by insulation sheets was held tightly using a belt clamp such that no air gaps existed, and heat losses could be minimized.

The samples also consisted of woven copper meshes embedded into them during the casting procedure to connect them to the data acquisition unit for measuring voltage difference and resistance. The copper meshes were made of copper wire of 300 µm diameter. The Seebeck coefficient tests were carried out by measuring the voltage difference between the two copper meshes in the sample. The temperature difference considered for measuring the Seebeck coefficient was measured at the same point where the voltage difference was measured. The data acquisition was carried out by connecting the electrical wires and the temperature sensors to a digital multimeter combined with a data logging and acquisition unit (Keithley’s DAQ6510).

#### 2.3.2. Electrical Conductivity Test

**Figure 2:**
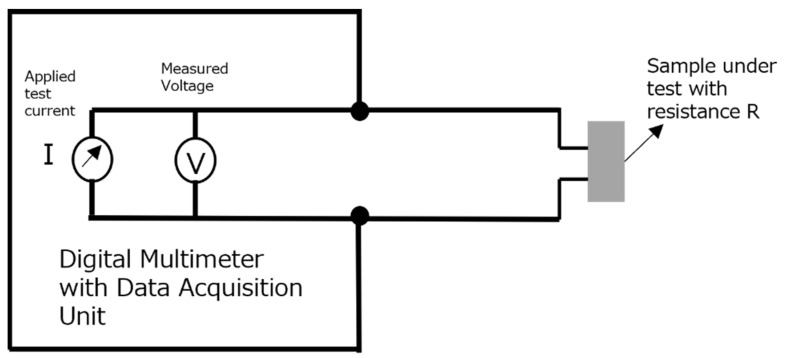
The 2W DC resistance measurement method.

The electrical resistance of the sample was determined using the two-wire DC method. The samples were connected to a digital multimeter and data acquisition unit using the copper meshes embedded in the cement samples. The electrical connections were made by soldering tinned copper wires (high-temperature resistant) with the copper meshes. The tinned copper wires on the other end were connected to the data logging unit. Electrical conductivity was derived by measuring electrical resistance of the sample and obtaining its resistivity by considering its geometric factor (length and cross-sectional area). The inverse of electrical resistivity led to the conductivity values for the sample. The circuit diagram of the DC resistance measurement for the sample is shown in Figure 2. Later, an electrical conductivity test was also performed by subjecting the sample to elevated temperatures, and the setup utilized for Seebeck coefficient tests was utilized for this purpose as well.

#### 2.3.3. Thermal Conductivity Test

**Figure 3:**
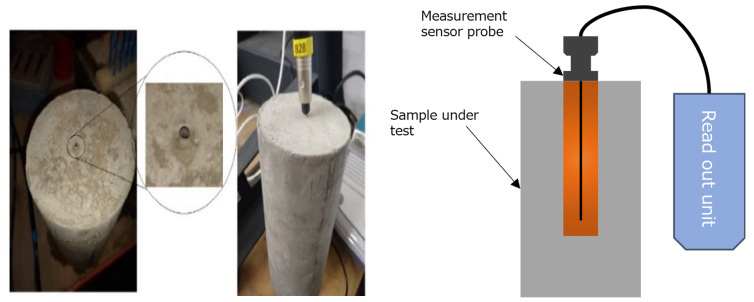
Cylindrical sample with sleeve used for thermal conductivity tests.

The Thermal Conductivity (TC) tests were carried out using equipment based on the Transient Line Source (TLS) method. While preparing samples for these tests, a cylindrical sleeve (100 mm long and 2 mm diameter) of a size that could accommodate the measuring probe of the THERMTEST TLS-100 thermal conductivity and resistivity meter was inserted in the sample. A photograph of the inserted sleeve in the cylindrical sample along with the thermal conductivity being measured is shown in Figure 3. The measurements were carried out at room temperature and TC tests were repeated 10 times for each sample and the average of the measured values was taken as the final value. This was done to ensure repeatability of the TC values while adapting a transient measurement technique.

## 3. Results

### 3.1. Seebeck Coefficient Test

**Figure 4:**
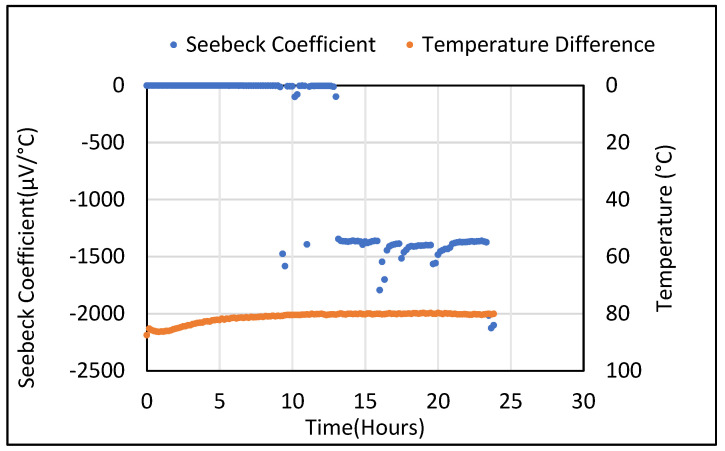
Seebeck coefficient of control sample at fixed temperature difference.

Figure 4 presents the initial Seebeck coefficient results from the plain cement samples described above with a constant temperature gradient applied. It was observed that a small DC voltage was recorded even though no temperature gradient was applied across the sample. The Seebeck voltages generated from a plain cement sample are found be in the range of 1 × 10^−5^–1 × 10^−6^ µV/°C. At approximately 13 h, a significantly higher Seebeck coefficient was recorded despite no change in temperature and this was also observed in other plain cement samples. The accuracy of the DAQ6510 multimeter and data acquisition unit is 0.0025% and the sensitivity is 100 nV (0.1 µV).

**Figure 5:**
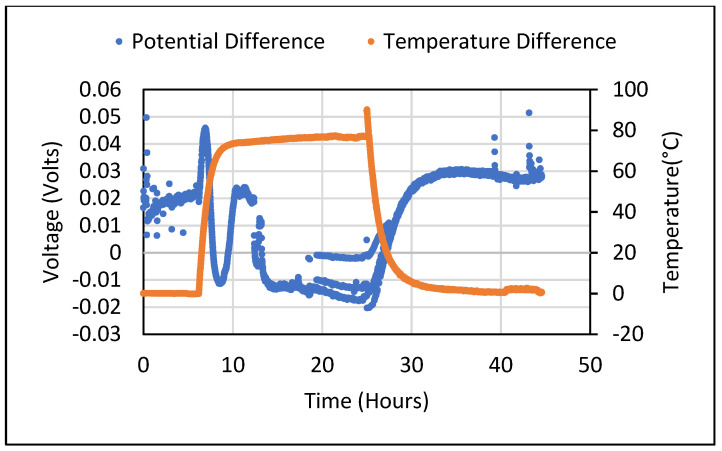
Voltage difference obtained from 5%wt. Bi_2_O_3_ cement composite at constant temperature difference.

**Figure 6:**
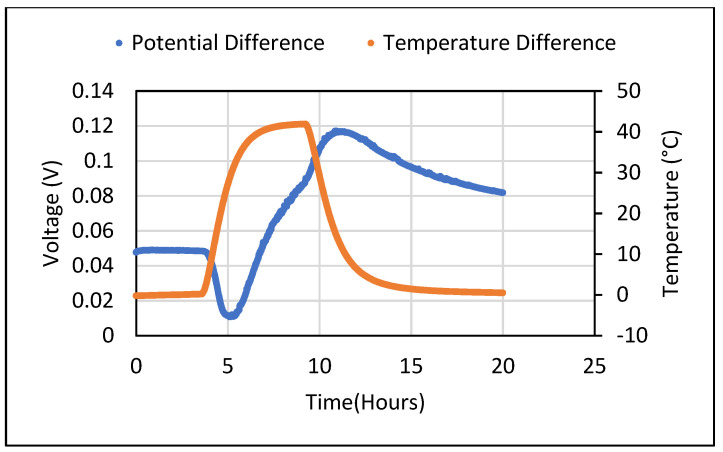
Potential difference obtained from a 5 wt.% Fe_2_O_3_ sample in saturated condition with changing temperature gradient.

Thereafter, the Seebeck coefficient tests were also carried out for the cement composites containing 5 wt.% of Bi_2_O_3_ and 5 wt.% of Fe_2_O_3_. For all tests carried out, samples in their saturated state produced a DC voltage in the range of millivolts despite not being subjected to any temperature gradient. When a constant temperature gradient was established across the sample, the obtained Seebeck voltage showed an odd sinusoidal pattern which shifted from positive to negative values during the temperature rise and fall taking place at the ends of the sample. The sinusoidal pattern of generated voltage for cement composites containing 5% wt. of Bi_2_O_3_ and 5% wt. of Fe_2_O_3_ is shown in Figure 5 and Figure 6**,** respectively. This pattern was repeatedly observed, especially when a rise in the temperature of the sample took place due to being subjected to heating. These ambiguous results were unable to give an idea about the real value of the Seebeck coefficient that could be obtained from the metal oxide-containing cement composites. Hence, a thorough analysis was carried out to find the source of error and mitigate it. The process used for doing so is described in detail in Section 4.

### 3.2. Electrical Conductivity Test

**Figure 7:**
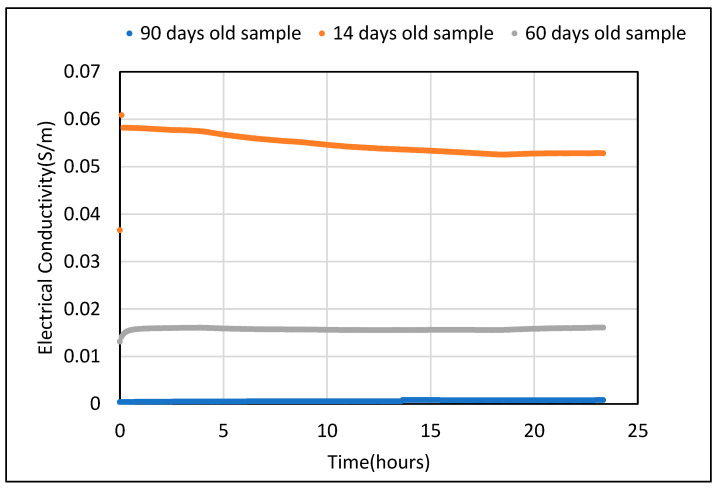
Electrical conductivity of control sample with 0.45 w/c after 14, 60 and 90 days.

The initial sets of Electrical Conductivity (EC) tests were carried out on the control sample at room temperature, without subjecting it to a temperature gradient. The EC value observed for a saturated control sample was found to be 0.07 S/m while, after drying (for 24 h at 105 °C), it reduced drastically to 2 × 10^−4^ S/m. Tests were simultaneously carried out for three different samples made and cured in similar conditions as mentioned. After allowing the samples to cure in a water tank for 7 days, they were subjected to ambient temperature and humidity conditions for 14, 60 and 90 days. Conductivity values were measured over a period of 24 h. As expected, the EC value for the 14-day-old sample was the highest at 0.06 S/m. Conductivity decreased with age and was observed to be 0.016 and 4 × 10^−4^ S/m for 60- and 90-day-old samples, respectively. The resulting values of electrical conductivity over 24 h for all three samples are shown in Figure 7.

**Figure 8:**
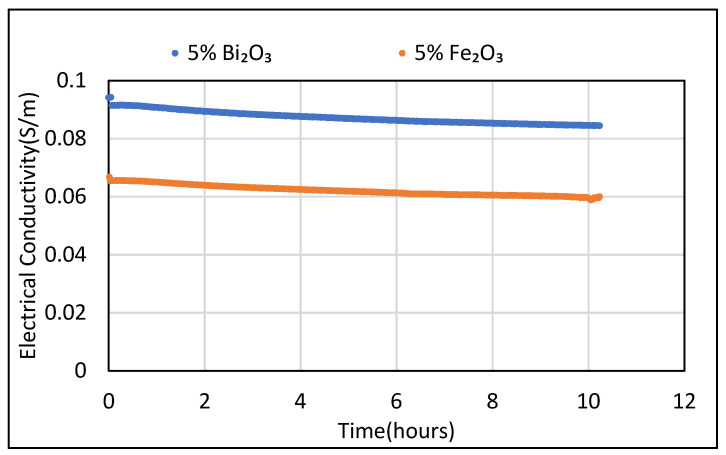
Electrical conductivity of cement composite with 5 wt.% Bi_2_O_3_ and 5wt.% Fe_2_O_3_ in saturated condition.

Similarly, the EC tests for cement composites with 5 wt.% Bi_2_O_3_ and 5 wt.% Fe_2_O_3_ in saturated conditions were also carried out at room temperature. The conductivity values for cement composite with 5% Bi_2_O_3_ displayed the highest conductivity value of 0.09 S/m, followed by the control sample and then the 5% Fe_2_O_3_ cement composite with EC values of 0.07 and 0.06 S/m, respectively. Conductivity values were found to decrease gradually with time for all measurements carried out. It was clear from the tests that, initially, due to a higher level of moisture present in the samples, the electrical conductivity is comparatively higher. Loss of moisture as time passes leads to reduction in conducting species in the sample matrix, which leads to reduction in conductivity values over time. The electrical conductivity values were still found to fall in the range of conductivity found in semiconductors [39,40,41]. Figure 8 represents the electrical conductivity values for 5 wt.% Bi_2_O_3_ and 5 wt.% Fe_2_O_3_ cement composites over a 10 h period of time.

Ideally, for characterizing the electrical properties for a material to be used as a thermoelectric material, its conductivity needs to be measured at elevated temperatures by subjecting the sample to varying temperature gradients. This has been carried out for ferrous oxide samples and the observations are mentioned below. During DC resistance measurement at room temperature, there was a voltage detected in the sample in the range of a few hundred millivolts interfering with the resistance measurements. This value reduced when the sample was dried as well as during its natural transition from a saturated to dry state. However, drying will lead to a drop in conductivity in cementitious materials containing composite materials [40,41].

### 3.3. Thermal Conductivity Test

**Figure 9:**
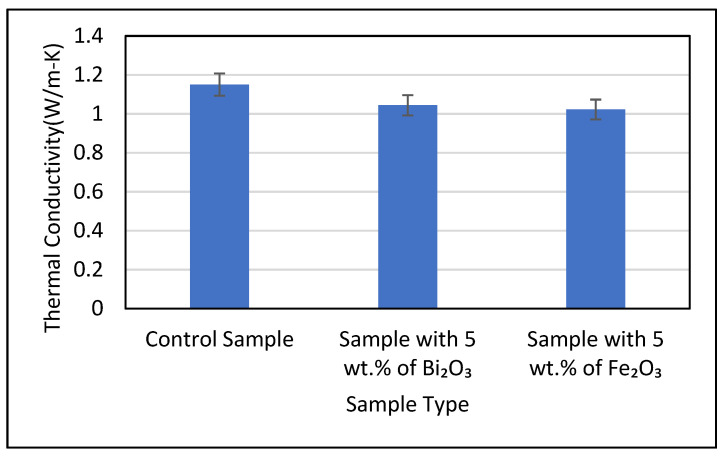
Thermal conductivity of control sample and cement composites with 5 wt.% Bi_2_O_3_ and 5 wt.% Fe_2_O_3_ at room temperature.

During the thermal conductivity measurements, it was made sure that the instrument used was in thermal equilibrium with the sample before each test was carried out. The TC of the control sample along with cement samples containing 5 wt.% of Bi_2_O_3_ and Fe_2_O_3_ was found to be 1.15, 1.044 and 1.022 W/m-K, respectively. The samples used for the measurements were in a saturated condition. The coefficients of variation observed for the measurements were found to be 1.59%, 1.88% and 1.47%, respectively. It was noted that the addition of metal oxides in the cement matrix led to a slight reduction in TC values compared to the control sample. The Bi_2_O_3_ and Fe_2_O_3_ cement composites saw a reduction of 9.2% and 11.1%, respectively, in their thermal conductivity values as compared to the TC of the control sample. The mean value obtained from thermal conductivity tests for all three samples is depicted in Figure 9.

## 4. Discussion

Due to the inconsistent results achieved with Seebeck coefficient tests, a thorough investigation was carried out to find the source of error that was leading to erroneous results in the measurement process. The first possible source of error studied was the instrument used to measure the voltage generated from the sample. Keithley’s DAQ6510, which was used for the data measurement and logging, was able to measure voltage in the microvolt scale with a sensitivity of 100 nV and an accuracy of 0.0025%. First, its offset voltage was determined. After disconnecting it from all circuits, the test lead wires were shorted together to find if the meter showed zero volts or not. The procedure was carried out for four different cases and the voltage obtained was in the range of 1 × 10^−6^ and 1 × 10^−7^ volts for all of them. To avoid external electrical interference, a few additional components were added to the experimental setup. A 3 mm thick aluminum sheet covered with a neoprene rubber sheet was placed at the bottom of all the equipment which had a grounding connection for the instrument’s chassis to be connected to. As a result of this arrangement, all the components in the setup were placed very close to each other. The instrument required a warm-up time of 30 min before taking any measurements. However, despite this, the results achieved were unstable. It was decided to warm up the instrument till a steady temperature gradient was established with a sample. DC voltage measurements were also carried out for samples without subjecting them to a temperature gradient. To establish thermal equilibrium, the instrument was allowed to warm up for 24 h.

**Figure 10:**
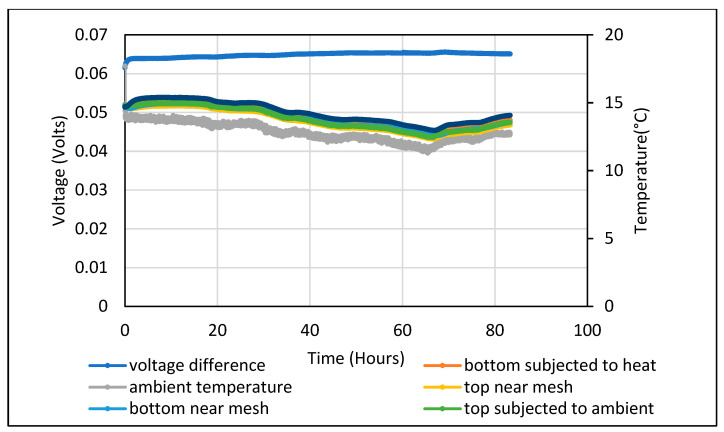
Potential difference obtained from a 5 wt.% Fe_2_O_3_ cement composite at zero temperature gradient.

**Figure 11:**
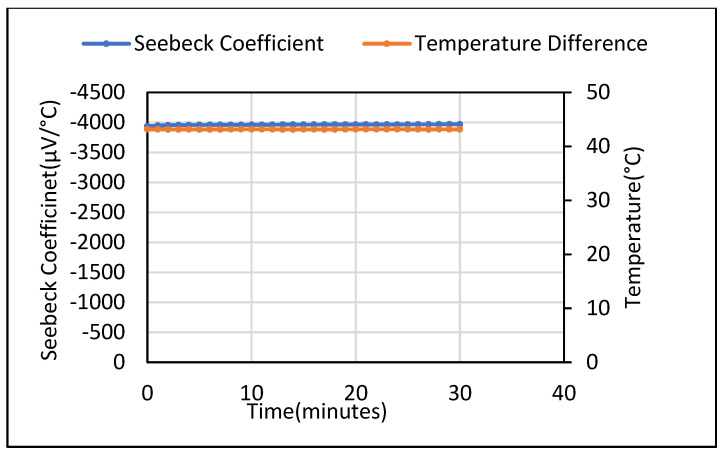
Seebeck coefficient for saturated 5 wt.% Fe_2_O_3_ cement composites for 30 min time period.

A steady 65–65 mV DC voltage was measured for the 5 wt.% Fe_2_O_3_ with no temperature gradient applied, as shown in Figure 10. The Seebeck coefficient obtained when a steady temperature gradient of 45 °C was obtained is shown in Figure 11. The measurements were taken over a 10-day period which yielded a gradually reducing voltage of 170–130 mV.

**Figure 12:**
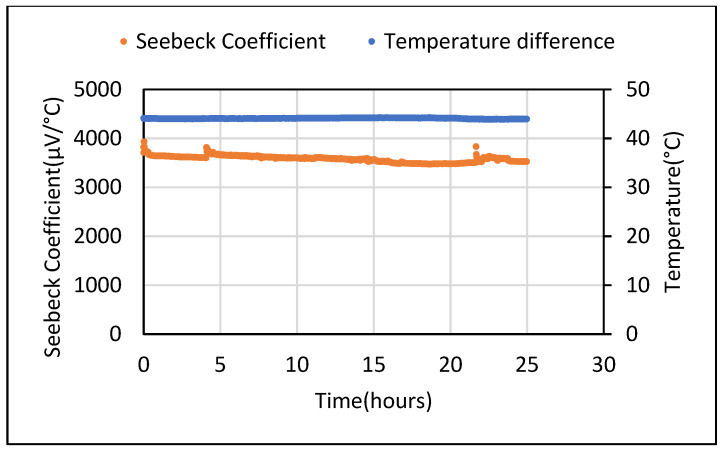
Seebeck coefficient at fixed temperature difference over 1-day time period for 5% Fe_2_O_3_ cement composite.

The voltage obtained was measured in both directions, i.e., from the hot to cold end and vice versa. The magnitude of voltage from both directions was found to be the same. The hot to cold voltage values showed a positive sign while the reverse order showed a negative sign. These results using the instrument were confirmed using two multimeters which give confidence that the sources of error have been eliminated. Another set of tests carried out from the hot to cold (positive) end for 24 h is shown in Figure 12.

**Figure 13:**
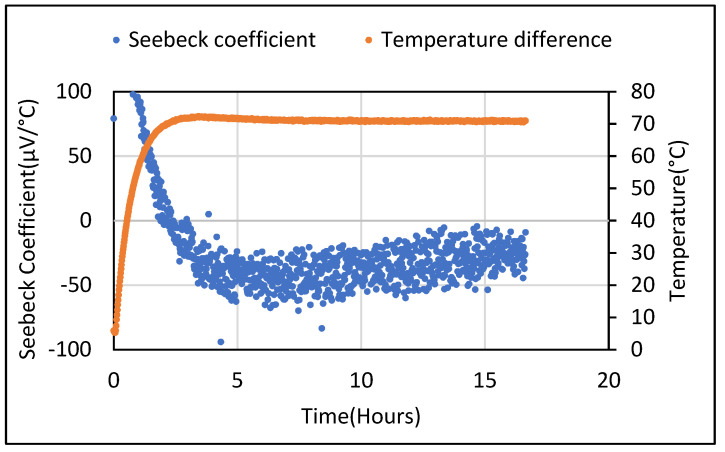
Seebeck coefficient obtained from dried 5 wt.% Bi_2_O_3_ sample.

**Figure 14:**
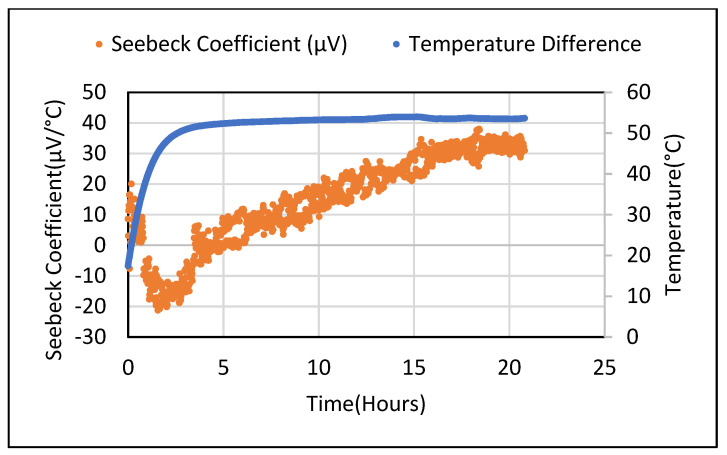
Seebeck coefficient values after drying the 5 wt.% Fe_2_O_3_ sample in oven for 24 h.

**Figure 15:**
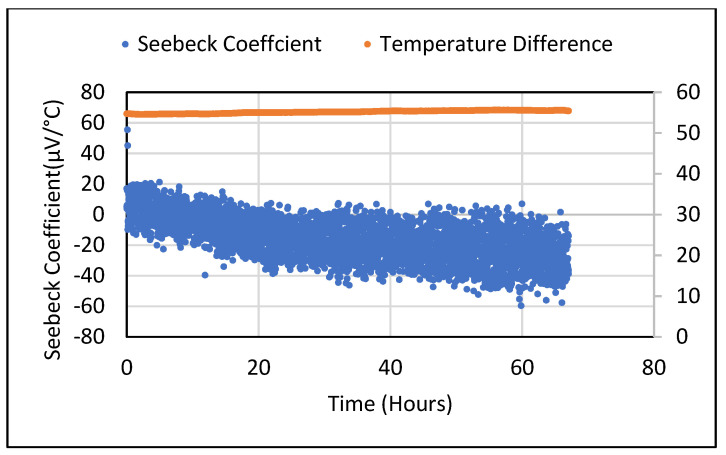
Seebeck coefficient values for 5 wt.% Fe_2_O_3_ at constant temperature gradient.

Seebeck tests were repeated for the saturated 5 wt.% Fe_2_O_3_ sample for different temperature gradients. They were obtained by manually adjusting the voltage supplied to the heating plate. For the 30–50 °C range (with a 5 °C step), a proportional nature of the obtained Seebeck voltage values was observed. The Seebeck values from the 5 wt.% Bi_2_O_3_ and Fe_2_O_3_ cement composites following drying were found to be lower and similar to the metal oxide samples. When these samples were placed in an ambient environment, an increase in moisture content was observed. Figure 13 shows the Seebeck coefficient obtained from dried 5 wt.% Bi_2_O_3_ cement composite which yielded negative readings (+80 to −80 µV/°C) when a steady state was achieved. For the 5 wt.% Fe_2_O_3_ material, the Seebeck coefficient varied as the temperature rose from −20 µV to +30 µV when stabilized, as shown in Figure 14. Figure 15 shows these values varying from +20 to −60 µV/°C over a 68 h period.

Thus, it can be observed that the Seebeck coefficient observed still fluctuates between positive and negative values in dried samples. This was not the case in saturated samples. An electrical conductivity test was carried out for a 5 wt.% of Fe_3_O_3_ cement composite in a saturated condition by subjecting it to a temperature gradient. The setup used for the Seebeck coefficient test was used for this purpose where, instead of DC voltage, two-wire DC resistance was measured. The conductivity results were obtained over a 2-day time period.

**Figure 16:**
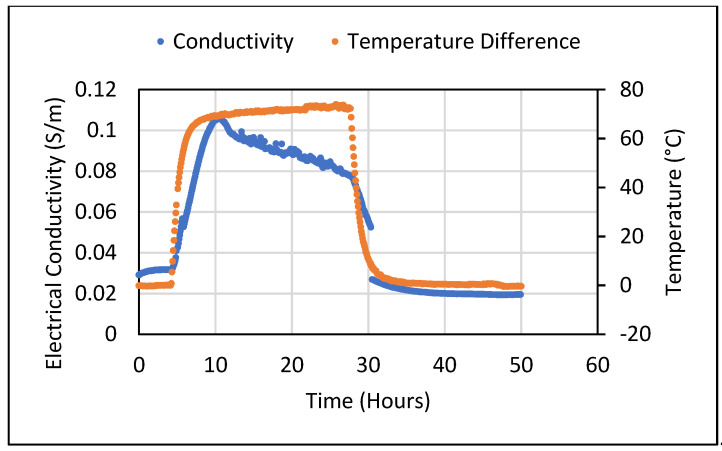
Electrical conductivity at elevated temperature for 5 wt.% Fe_2_O_3_ cement composite.

The electrical conductivity values showed a drastic rise when a temperature gradient was applied across the sample. This could be attributed to a temperature rise leading to increased vibrations resulting in the scattering of electrons. Scattering can change its mean free path which represents the electrons’ ability to travel without scattering which ultimately results in increased electrical conductivity. When the temperature gradient no longer existed, the conductivity values were observed to fall back to the original values observed when the gradient was not applied. The graph showing the change in electrical conductivity when a temperature gradient is applied across a 5 wt.% Fe_2_O_3_ sample is depicted in Figure 16.

An important fact observed while carrying out the electrical conductivity tests at elevated temperatures was the presence of thermoelectric emfs in the circuit. When resistance is measured at elevated temperatures there is a significant amount of DC voltage observed because of two different parts of the circuit being at different temperatures. When DC resistance of the sample is measured by an instrument, it supplies a current of a known value and measures the drop in the voltage due to the sample’s resistance. This DC voltage is measured and used to calculate the resistance value of the sample in the background. However, the voltage measured from the sample (*V_total_*) is found to be a combined effect of Seebeck voltage (*α*Δ*T*) and resistive voltage (*V_res_*) as represented in Equation (2) [42].

(2) $$V_{total}=\alpha \Delta T+V_{res}.$$

The values of resistive voltages and Seebeck voltages are in the same range for semiconductor materials [42]. Thus, an undesirable Seebeck effect caused by the applied temperature gradient interferes with the measurement process. This fact makes DC resistance measurement unfit for high-temperature resistance measurement. This effect can be eliminated by using AC resistance measurement or by including a fast-switching device in the DC resistance measuring setup. Other factors that could affect the measured DC voltage for Seebeck tests includes the thermoelectric emfs resulting in different parts of the circuit being at different temperatures. A junction of copper and tinned copper could lead to a thermo emf of 1–3 µV/°C. It is important to protect the junctions from being subjected to oxidation as a copper–copper oxide junction could generate an emf of approximately 1000 µV/°C in magnitude [42]. This could add up in the DC voltages measured by the instrument and lead to ambiguous results.

## 5. Conclusions

This work presented Seebeck coefficient, DC voltage and resistance amd thermal and electrical conductivity measurements taken on cements doped with two metal oxide powders and their effect on the materials’ performance as a TE element. This work found that, regardless of the metal oxide added, the Seebeck coefficient and electrical conductivity are reduced when dried. Further measurements were possible only when the moisture contribution was eliminated. In terms of thermal conductivity, the control sample demonstrated higher values than both enhanced cements. Finally, it was found that the method used to measure DC resistance here was unfit for use due to interferences generated.

It is clear that the age and moisture content have a significant role in the thermoelectric performance and behavior of cement-based composites. As a result, further work is required to develop an automatically controlled experimental setup to take stable measurements using variable temperature gradients.

## Figures and Tables

**Table 1:** Comparison of characterization techniques and methods used for thermoelectric characterization of cement-based materials developed to date in the literature.

Sr No.	Sample Details	Electrical Conductivity	Seebeck Coefficient	Thermal Conductivity	Reference
**1**	Carbon fiber-reinforced cement 40 × 40 × 40 mm	Two-wire DC method using multimeter	Potentiometer measured voltage and thermostats achieved and measured ∆T	Not measured	[17]
**2**	1. Carbon fiber-reinforced cement 75 × 15 × 15 mm 2. Bromine intercalated CFRC 75 × 15 × 15 mm	Not measured	Hot side: Resistance plate heater (up to 65 °C) Cold side: Close to room temperature Contacts: Silver paste, copper foil and copper wire Instrument: Keithley Multimeter	Not measured	[30,31]
**3**	Carbon fiber -reinforced cement along with silica fume and fly ash 100 × 100 × 100 mm	Not measured	Thermocouples measured temperature gradient Multimeter measured Seebeck voltage	Not measured	[32]
**4**	Carbon fiber-reinforced cement	Four-wire DC using multimeter Sample: 160 × 40 × 40 mm	Hot side: Ceramic resistance heater (up to 90 °C) Cold side: At room temperature (maintained at 25 °C) Contacts: Copper plate and copper wire Instrument: Fluke B15 multimeter Sample: 160 × 40 × 40 mm	Steady state method in thermal Conductometer sample: 180 mm diameter and 20 mm height	[33]
**5**	Carbon Nanotube-reinforced cement composite	Four-wire DC using multimeter Sample: 10 × 10 × 40 mm	Hot side: Resistance plate heater (up to 100 °C) Cold side: 5 °C higher than hot side temperature (starting at 30 °C) Contacts: Silver paste and copper wire Instrument: T type thermocouple and multimeter Sample: 10 × 10 × 40 mm	Laser flash diffusion analysis sample: 12.7 mm diameter and 1.0–3.0 mm height Measured for 3 samples and averaged values considered	[34]
**6**	P- and N-doped carbon nanotube-enhanced cement composite 60 × 10 × 10 mm	Two-wire DC using multimeter	Hot side: Resistance heater (40–50 °C) Cold side: At ambient temperature (25 °C) Contacts: Silver paste and copper wire Instrument: IR thermometer and multimeter	Not measured	[35]
**7**	Cement composite enhanced with expanded graphite and carbon fiber 40 × 10 × 10 mm	Four-probe DC method using silver paste and conductive wires as contact	Hot side: Resistance heater (33–80 °C) Cold side: Ambient side also heated Contacts: Silver paste and type T thermocouples for temperature Instrument: DMM and data Acquisition unit	Not measured	[36]
**8**	Cement composite enhanced with expanded graphite and carbon fiber 40 × 10 × 10 mm	Four-probe DC method using silver paste and conductive wires as contact using a DMM	Hot side: Ceramic resistance heater (30–100 °C) Cold side: Ambient side also heated (5 °C above hot side end) Contacts: Silver paste and type T thermocouples for temperature Instrument: DMM and data acquisition unit	Laser flash diffusivity analysis sample: 12.7 mm diameter and 1.0 mm–3.0 mm height Measurements carried out at room Temperature	[24]
**9**	Graphene-enhanced cement composite 10 × 4 × 4 mm	Four-probe DC using RZ2001i Ozawa Science thermoelectric characterizing device	Steady DC method used for Seebeck coefficient measurement Range: Room temperature to 75 °C	DSC and LFA method used for thermal conductivity tests in inert environment (nitrogen gas was supplied) Range: 25–75 °C	[37]
**10**	Cement composite enhanced with stainless steel fibers 75 × 15 × 15 mm	Four-probe DC using multimeter Contacts: Copper foil, silver paint and copper wires	Hot side: Resistance plate heater (up to 85 °C) Contacts: Silver paste and copper wire Type T thermocouples for temperature Instrument: Keithley multimeter	Not measured	[38]
**11**	CFRC cement composite enhanced with Ca_3_Co_4_O_9_ 160 × 40 × 40 mm	Not measured	Hot side: Resistance plate heater Cold side: Maintained at room temperature Contacts: Copper plates and copper wires Instrument: Fluke B15 multimeter with a voltage amplifier	Not measured	[21]
**12**	CFRC cement composite enhanced with metal oxides Bi_2_O_3_ and Fe_2_O_3_ 160 × 40 × 40 mm	Not measured	Hot side: Resistance plate heater (up to 90 °C) Cold side: Maintained at room temperature Contacts: Not mentioned Instrument: Fluke B15 multimeter with a voltage amplifier	Not measured	[19]
**13**	Cement composites enhanced with pyrolytic carbon fiber and Fe_2_O_3_ 10 × 10 × 40 mm	Four-probe DC method using silver paste and conductive wires as contacts	Hot side: Resistance heater (35–80 °C), gradient of 5 °C was maintained Cold side: Maintained at room temperature Contacts: Silver paste Instrument: Thermocouple and multimeter	Determined theoretically using assumed values	[13]
**14**	Cement composites enhanced with ZnO and α-Fe_2_O_3_ nanopowders 40 × 40 × 160 mm	Four-probe DC using copper wires and silver paste as contacts Instrument: Fluke B15 multimeter	Hot side: Resistance plate heater (up to 70 °C) Cold side: At room temperature by contact with flowing water Contacts: Copper plate Instrument: K type thermocouple and Fluke B15 multimeter	Steady state method used for measurement where cold side was kept at 20 °C and hot side at 70 °C Sample size 300 × 300 × 20 mm	[20]
**15**	MgO_2_-enhanced cement composites 40 × 40 × 160 mm	Four-probe DC embedded copper meshes and silver adhesives used for contact	Hot side: Resistance heater up to 60 °C (temperature gradients of up to 50 °C) Cold side: At room temperature Contacts: Copper plate, conductive wires and silver paper Instrument: K type thermocouple and Fluke 289C multimeter	Steady state thermal conductivity tester used for a sample of 130 mm diameter and 40 mm height	[22]
**16**	Cement composites enhanced with ZnO and Al-doped ZnO powders	Two-probe AC impedance measurement for cylindrical samples of 70 mm height and 35 mm diameter	Hot side: Resistance heater up to 85 °C Cold side: At room temperature (23 ± 2 °C) Contacts: Copper plates and copper wires Instrument: Omega CN616 temperature controller and Keithley multimeter 40 × 40 mm of surface area	Longitudinal guarded comparative calorimeter used for cylindrical samples of 25.4 mm diameter and 50.8 mm height	[23]
**17**	Graphene- and nano-ZnO-enhanced cement composites	Four-probe DC using RZ20001i Ozawa Science thermoelectric characterizing device 4 × 4 × 10 mm	Steady state DC method used for Seebeck coefficient measurement Range: Room temperature to 75 °C 4 × 4 × 10 mm	Laser flash diffusivity analysis and differential scanning calorimeter used for sample having 17 mm diameter and 2 mm height	[32]

**Table 2:** CEM I cement chemical composition as provided by Irish Cements.

Contents	Percentage (%)
SiO_2_	18.29%
Al_2_O_3_	5.08%
Fe_2_O_3_	2.78%
CaO	63.89%
SO_3_	2.64%
F. Cao	1.57%
Loss on Ignition (LOI)	2.79%
Na_2_O Eq. (Alkali Equivalent)	0.59%

Here, the chloride content of the cement is not included as it was not available from the reports.

## Data Availability

Not applicable.
